# Retraction: The improvement of large High-Density Lipoprotein (HDL) particle levels, and presumably HDL metabolism, depend on effects of low-carbohydrate diet and weight loss

**DOI:** 10.17179/excli2016-570

**Published:** 2016-09-19

**Authors:** C. Finelli, P. Crispino, S. Gioia, N. La Sala, L. D'amico, M. La Grotta, O. Miro, D. Colarusso

**Affiliations:** 1 Center of Obesity and Eating Disorders, Stella Maris Mediterraneum Foundation, Chiaromonte, Potenza, Italy; 2 U.O.C. Medicina Interna, Urgenza ed Accettazione, P.O. S. Giovanni, Lagonegro - ASP Potenza

## ⁯

Dear editor,

As corresponding author I ask for retraction of our article Finelli et al. (2016[1]) with the consent of all co-authors, because of unauthorized reproduction of confidential content of another manuscript. The data in the retracted article actually are from a cohort of patients from the Boston, MA enrolled in a trial registered in ClinicalTrials.gov, NCT02454127. We deeply regret these circumstances and apologize to the scientific community.

Carmine Finelli, MD PhD
